# Postpartum modern contraception utilization and its determinants in Ethiopia: A systematic review and meta-analysis

**DOI:** 10.1371/journal.pone.0243776

**Published:** 2020-12-14

**Authors:** Bizuneh Wakuma, Getu Mosisa, Werku Etafa, Diriba Mulisa, Tadesse Tolossa, Getahun Fetensa, Merga Besho, Mohammed Gebre, Reta Tsegaye

**Affiliations:** 1 School of Nursing and Midwifery, Institutes of Health Sciences, Wollega University, Nekemte, Ethiopia; 2 Department of Public Health, Institutes of Health Sciences, Wollega University, Nekemte, Ethiopia; 3 Department of Pharmacy, Institute of Health Sciences, Wollega University, Nekemte, Ethiopia; James Madison University, UNITED STATES

## Abstract

**Background:**

Contraceptive use is the best and most cost-effective strategy to reduce feto-maternal adverse effects of short birth intervals. More than two-thirds of women in developing countries who do not want to conceive are not using contraception methods. Although there were various primary studies in different parts of the country, there is no nationally representative evidence on postpartum modern contraception utilization and its determinants in Ethiopia.

**Objective:**

This review was aimed to determine the best available pieces of evidence to pool the magnitude of postpartum modern contraception utilization and find out its determinants.

**Methods:**

Published studies were extensively searched by using electronic databases and unpublished studies were identified from the digital library. All observational studies conducted on the magnitude of postpartum modern contraception utilization and its determinants in Ethiopia were included. Data were extracted on the Microsoft Excel spreadsheet and analyzed using STATA 14.1 version. A random-effects model was used to estimate the pooled magnitude of postpartum modern contraception utilization with a 95% confidence interval (CI). Inverse variance (I2) was used to identify the presence of heterogeneity and forest plot was used to estimate the pooled magnitude of postpartum contraception utilization. The presence of publication bias was assessed by funnel plots and Egger’s statistical tests. Sub-group analysis was computed to minimize underlying heterogeneity.

**Findings:**

In this review, 19 primary studies were included. The pooled magnitude of postpartum modern contraception utilization in Ethiopia was 45.79% (95%CI 36.45%, 55.13%). The review found that having more than four Antenatal care visits(ANC), having postnatal care visit (PNC), having a formal education, history of family planning use, history of counseling on family planning, and having greater than four alive children as significant determinants of postpartum modern contraception utilization.

**Conclusion:**

The magnitude of postpartum modern contraception utilization in Ethiopia was low. ANC visit, PNC visit, maternal educational status, history of previous family planning use, counseling on family planning, and number of alive children were found to be significant determinants of postpartum modern contraception utilization. Therefore, strengthening focused ANC and PNC services to encourage women in utilizing modern contraception during the postnatal period is needed.

## Introduction

Family planning refers to a conscious effort by a couple to limit or space the number of children they have through the use of contraceptive methods [1]. Contraceptive methods are classified as modern or traditional. The intrauterine contraceptive device (IUD), implants, injectable, pills, and emergency contraception are among the modern contraceptives [1]. Postpartum contraception is the initiation of family planning services within the first 12 months following childbirth to prevent narrowly spaced and unintended pregnancies [2]. Improvement in contraceptive utilization contributed to fertility decline by three-fourth in the past six decades and reduction of pregnancy in high parity women, which eventually improved maternal survival rates in developing countries [3]. Postpartum use of family planning plays a crucial role in saving the lives of mothers and children. However, postpartum women are among those with a greater unmet need for family planning [4]. In Ethiopia in the year 2016, about 22% of postpartum women have an unmet need for family planning [5]. The unique family planning needs of postpartum women might be because of breastfeeding practice of postpartum women that determines the duration of amenorrhea they had experienced. Women who do not practice exclusive breastfeeding following birth may get pregnant before menses returned [4].

Maternal mortality continues to be a major public health challenge worldwide, particularly in developing countries. Globally, for the year 2017, maternal death is estimated to be 295,000 among which 94% occurred in low resource settings. Sub-Saharan Africa accounts for approximately 66% of maternal death when the majority of their causes are preventable [6]. Contraceptive use has reduced maternal death by 40% in the last 25 years worldwide and a further 30% of maternal death would fall if all women who want to avoid pregnancy use an effective contraceptive method in developing countries [3]. Available evidence indicates that short interpregnancy interval has adverse feto-maternal outcome [7], as it ends with miscarriage, stillbirth, induced abortion, prematurity, low birth weight, newborn and maternal death [3, 7, 8]. Contraceptive use improves birth interval length and this in turn improves under-five survival. For instance, spacing pregnancy for 24 and 36 months to conceive, would fall under-five mortality by 13% and 25% respectively [9]. An easy way for lowering under-five mortality in Ethiopia is promoting optimum birth interval which can be achieved by encouraging contraceptive use [10].

Although Family planning 2020 has made remarkable progress about solving the issue of unmet need for family planning, 70% of women in a developing country who do not want to conceive are not using it [11]. Despite contraceptive use is among the best and most cost-effective strategy to reduce feto-maternal adverse effects of the short interpregnancy interval, still, there is low utilization [9, 11]. Reducing chronic malnutrition among children, increasing female adolescent school attendance rates through preventing unwanted & unplanned pregnancy, and reducing abortion rates are among some of the merits of contraceptive use [7, 9, 12, 13]. The pregnancy-related maternal mortality ratio in Ethiopia was 412 per 100,000 live births [5]. Ethiopia has a larger share of the World maternal death as 14,000 maternal deaths reported as of the year 2017; hence Ethiopia has vast homework to achieve SDG4 & 5 by 2030 [6]. The magnitude of postpartum modern contraception utilization in Ethiopia ranges from 12.05% to 80.32% [14, 15]. Different studies found antenatal care, postnatal care, resumed sexual activity, partner approval for contraceptive use, menses return, and counseling on family planning use as determinants of postpartum modern contraception use [15–20].

Though there were various primary studies done in different regions of the country, there is no nationally representative evidence on the magnitude of postpartum modern contraception utilization and the pooled effects of its determinants in Ethiopia. Therefore, this review was aimed to determine the best available evidence to pool the Magnitude of postpartum modern contraception utilization and find out its determinants. The finding of this study will provide scientific evidence to inform the program planners and policymakers for service improvement. Moreover, it also helps the health care providers in providing scientific evidences for practice.

## Methods

### Search strategy

Preferred Reporting Items of Systematic Reviews and Meta-Analysis (PRISMA) checklist guidelines were used to ensure scientific consistency [21]. The review was conducted to estimate the pooled magnitude of postpartum modern contraception utilization and its determinants in Ethiopia. The review protocol was registered on Prospero with the registration number of CRD42019130288 to avoid duplication of a similar review. Furthermore, the presence of the existing systematic reviews and meta-analysis was checked by using the Cochrane library. Extensive database searching such as; Medline, Scopes, PubMed, CINAHL, Embase, Cochrane library, the JBI Library, the Web of Science, Google Scholar, and Google search engines were included in the review. All Articles were accessed using the following search terms "Contraception, Family planning, Contraceptive, Utilization, Use, Utilization, Postpartum mothers, Postpartum period, Post-delivery, puerperal period, Ethiopia”. The key terms were used individually and in combination through “AND” and “OR” boolean operators. The search for all articles was conducted from November 14th to December 5th, 2019 without limiting the publication dates of the articles.

The full search string for Pubmed database was "Search ((((((("Contraception"[Mesh] OR "Contraception, Postcoital"[Mesh] OR "Contraception, Barrier"[Mesh] OR "Contraception, Immunologic"[Mesh] OR "Contraception Behavior"[Mesh] OR "Long-Acting Reversible Contraception"[Mesh] OR "Contraceptive Effectiveness"[Mesh])) OR (Contraception [tw] OR Family planning [tw]))) AND ((("Patient Acceptance of Health Care"[Mesh] OR "Facilities and Services Utilization"[Mesh])) OR (Uptake [tw] OR Use [tw] OR Utilization [tw]))) AND ((("Postpartum Period"[Mesh] OR "Postnatal Care"[Mesh] OR "Sexual Abstinence"[Mesh])) OR (Postpartum [tw] OR Post delivery [tw] OR Puerperal period [tw])))) AND Ethiopia".

### Eligibility criteria

This review included only studies conducted in Ethiopia that determined the magnitude of postpartum modern contraception utilization and its determinants. Both published and unpublished articles were searched and screened for inclusion. This review included all observational study designs (cross-sectional studies, case-control studies, and cohort studies). Moreover, articles that were only published in the English language were included. All studies that were published in the form of journal articles, master’s thesis, and dissertation till the final date of data analysis were included. Studies with the methodological problems and review articles were excluded from the analysis. Duplicate reports and inconsistent outcome measures were excluded. This systematic review and meta-analysis used the COCOPOP (Condition, Context, and Population) framework to assess the eligibility of the articles included. The study Population (POP) was postpartum women, the Condition (CO) was the Magnitude of modern postpartum contraception utilization, and the context (CO) only studies conducted in Ethiopia.

**Fig 1:**
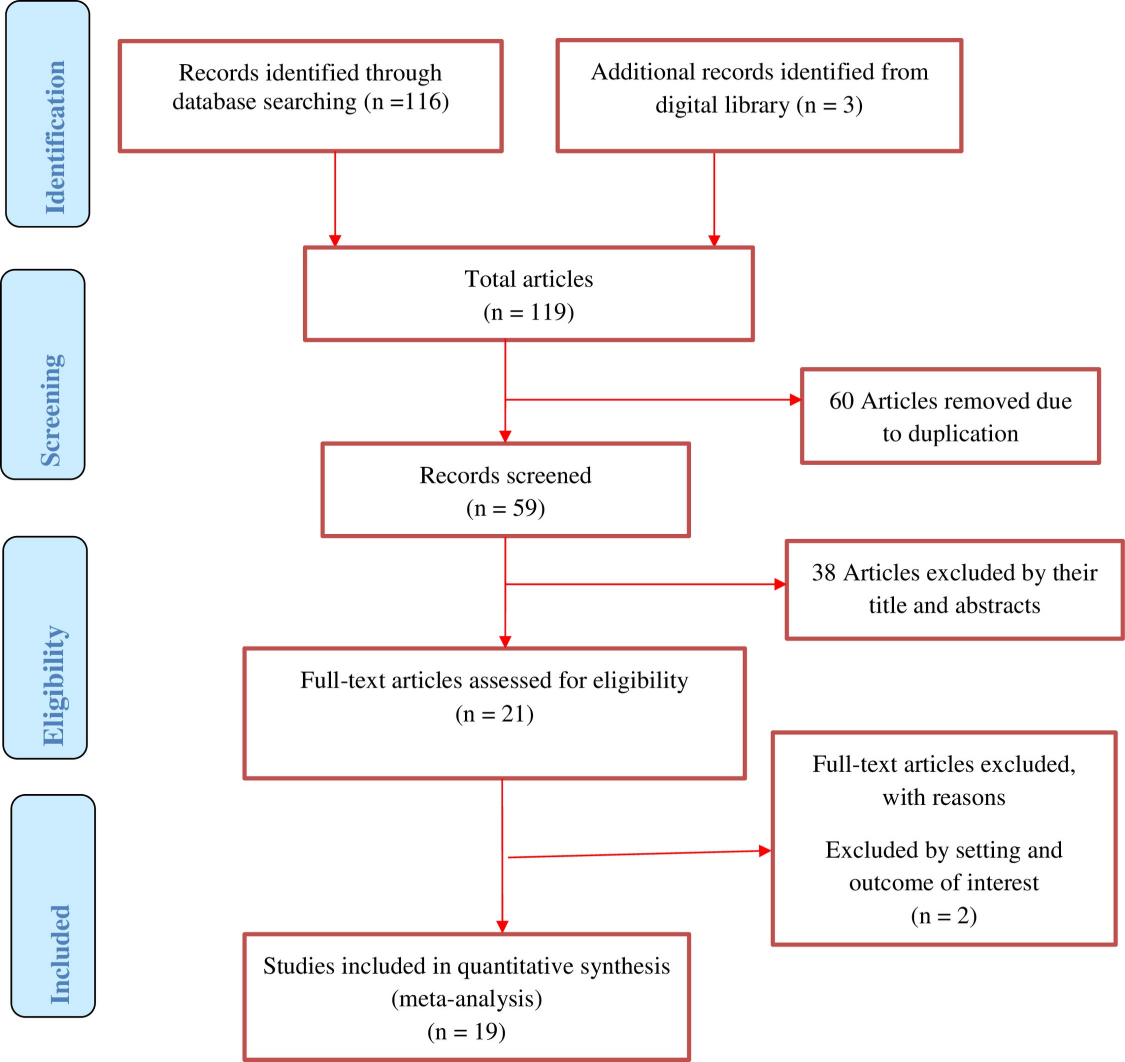
PRISMA flow diagram of included studies in the systematic review and meta-analysis postpartum modern contraception utilization in Ethiopia, 2020.

At the beginning of our search, 119 articles were found, of which 60 were removed due to duplicates and the rest 59 articles were screened for eligibility. From 59 articles 38 were excluded by title and abstracts and 21 articles assessed for full text among which two articles excluded because of non-relevance to the current review and the remaining 19 articles were included in the final meta-analysis (Fig 1).

### Study outcome

The outcome of this systematic review and meta-analysis was the pooled magnitude and determinants of postpartum modern contraception utilization. Postpartum contraception is defined as the initiation of family planning services within the first 12 months following childbirth to prevent closely spaced and unintended pregnancies [2]. The second outcome of this study was the determinants of postpartum modern contraception utilization. Data for this outcome was extracted in a format of 2x2 tables on the Microsoft Excel spreadsheet, and then the odds ratio for each factor was calculated. Determinant factors included in this review were the Number of ANC visit (four and greater versus less than four), PNC visit (PNC visit versus No PNC visit), Educational status (formal education versus No formal education), Parity (five and more versus less than five), Counselling on family planning (Counselled versus Not counseled), previous family planning use (Previous use versus no previous use), Partner approval (partner approved versus partner not approved), Place of residence (Urban versus Rural)and Number of alive children(less than four versus four and greater).

### Quality assessment and data extraction

The Joanna Briggs Institute Meta-Analysis of Statistics Assessment and Review Instrument (JBI-MAStARI) was used for quality appraisal [22]. Data were extracted by two data extractors (BW and GM) using a standardized data extraction checklist on Microsoft excels spreadsheet. This systematic review followed the Preferred Reporting Items for Systematic Review and Meta-Analyses (PRISMA) flow chart to identify and select relevant studies for this analysis. Initially, duplicated kinds of literature were excluded by using Endnotes version 7.2, a citation manager. The studies were excluded by reviewing their titles and abstracts. Full-text articles or reports were evaluated for the remaining literature. Based on the preset inclusion and exclusion criteria, eligibility of the studies was assessed. For the outcome variable (postpartum contraception utilization) and exposure variables (Associated factors), data were extracted in a format of two by two tables, and then the log OR was calculated based on the findings of the original studies. The checklist for data extraction contains the name of authors, publication year, region (the area where studies were conducted), study design, the magnitude of postpartum contraception utilization, sample size, and response rate (Table 1). Each article was checked by two authors independently (BW and GM).

**Table 1:** Descriptive summary of 19 included articles to pool magnitude of postpartum modern contraception utilization in Ethiopia, 2020.

Authors	Year	Region	Design	Study area	Sample size	Magnitude
Gonie, A et al. [32]	2018	Oromia	Cross-sectional	Bale	660	41.52(37.76,45.27)
Teka, T et al. [20]	2018	Oromia	Cross-sectional	Gida Ayana district	603	45.44(41.47,49.41)
Gebremedhin, A et al. [14]	2018	Addis Ababa	Cross-sectional	Kolfe keranyo subcity	803	80.32(77.57,83.07)
Tedla, Z et al. [36]	2017	Addis Ababa	Cross-sectional	Addis A baba	631	50.24(46.34,54.14)
Nigussie, A et al [15]	2016	Somali	cross-sectional	Kebribeyah town	556	12.05(9.34,14.76)
Gejo, N et al. [35]	2019	SNNP	cross-sectional	Hossana town	368	73.64(69.14,78.14)
Abraha, T et al. [37]	2018	Tigray	cross-sectional	Central zone of Tigray	1109	38.32(35.46,41.18)
Gebremariam, A et al. [34]	2017	Tigray	cross-sectional	Ganta-Afeshum district	605	68.10(64.39;71.81)
Abraha, T et al. [33]	2017	Tigray	cross-sectional	Aksum	590	47.97(43.93,52.00)
Gizaw, W et al. [30]	2017	Amhara	cross-sectional	Gozamendistrict	829	46.68(43.29,50.08)
Berta, M et al [28]	2018	Amhara	cross-sectional	Gondar town	404	45.79(40.93,50.65)
Tafere, T et al [18]	2018	Amhara	Follow up study	Bahir dar city	970	16.19(13.87,18.50)
Wudineh, K et al [31]	2018	Amhara	cross-sectional	Debra tabor town	588	28.40(24.76,32.05)
Taye, E et al [19]	2019	Amhara	cross-sectional	Debra tabor town	563	61.10(57.07,65.13)
Derso, T et al [29]	2019	Amhara	cross-sectional	Gondar zuria district	603	43.78(39.82,47.74)
Abera, Y et al [38]	2015	Amhara	cross-sectional	Gondar town	705	48.23(44.54,51.92)
Demie, T et al. [25]	2018	Amhara	cross-sectional	DebreBerhan town	248	33.06(27.21,38.92)
Belete, G et al [27]	2019	Amhara	cross-sectional	Injibira town	402	58.21(53.39,63.03)
Dona, A et al [16]	2018	SNNP	cross-sectional	Aroressa district	695	31.22(27.78,34.67)
**Over all with weights from random effect**						45.79(36.45,55.13)

(AA-Addis Ababa, CI-Confidence Interval, SNNP-Southern Nation Nationalities and people).

Any form of disagreement was resolved by discussion between two independent reviewers and involving a third reviewer for consensus (WE).

### Statistical analysis

Data were extracted from each eligible article using a format prepared in Microsoft Excel spreadsheet. STATA/SE for windows version 14 software was used to calculate the pooled magnitude of postpartum modern contraception utilization with a 95% confidence level by using the Der Simonian and Laird random-effects meta-analysis (random-effects model). The logarithm and standard error of the odds ratio (OR) for each included study were generated using the “generate” command in STATA.

### Heterogeneity and publication bias

Heterogeneity across studies was assessed by determining the p-values of I^2^-test statistics. I^2^ test statistics values of 0, 25, 50, and 75% were considered as no, low, moderate, and high degrees of heterogeneity, respectively [23]. A funnel plot was used to check publication bias. Besides, Egger’s weighted regression test and Begg’s test were used to check publication bias [24]. A p-value of less than 0.05 was used to declare the statistical significance of publication bias.

## Results

### Description of characteristics of reviewed articles

Nineteen articles included in this systematic review and meta-analysis are summarized in Table 1. Eighteen articles were cross-sectional in design while one article was a follow-up study with a sample size ranging from 248 in Debre Berhan town [25] to 1109 in the Tigray region [26]. With regard to the region in which the study conducted, nine Articles were from the Amhara region [16, 18, 19, 25, 27–31], two from Oromia [19, 32], Three from Tigray [26, 33, 34] two from SNNP [16, 35], two from Addis Ababa [14, 36] and one from Somali [15] region with publication year ranging from 2015 to 2019 (Table 1).

### Magnitude of postpartum modern contraception utilization

**Fig 2:**
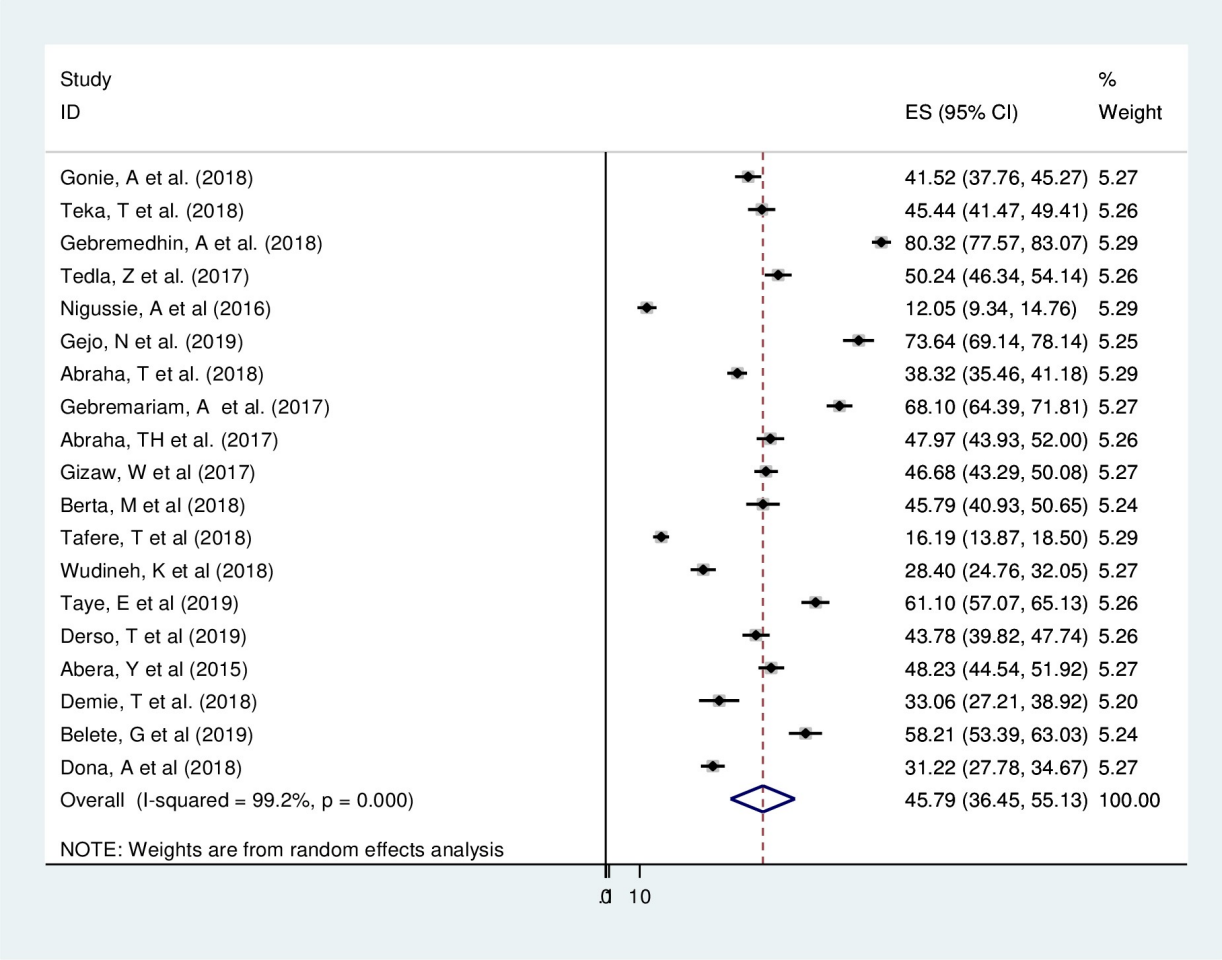
Forest plot of the pooled magnitude of postpartum modern contraception utilization in Ethiopia, 2020.

This review analyzed data from 11,932 postpartum women to estimate the pooled magnitude of postpartum modern contraception utilization. A total of 19(18 published and one unpublished) articles were included in this review yielding the pooled magnitude of postpartum modern contraception utilization in Ethiopia 45.79% (95% CI: 36.45%, 55.13%) with the lowest, 12.05% (95% CI: 9.34%, 14.76%) in Somali regional state [15] and the highest 80.32% (95% CI: 77.57%, 83.07%) in Addis Ababa [14] (Fig 2).

### Subgroup analysis

**Fig 3:**
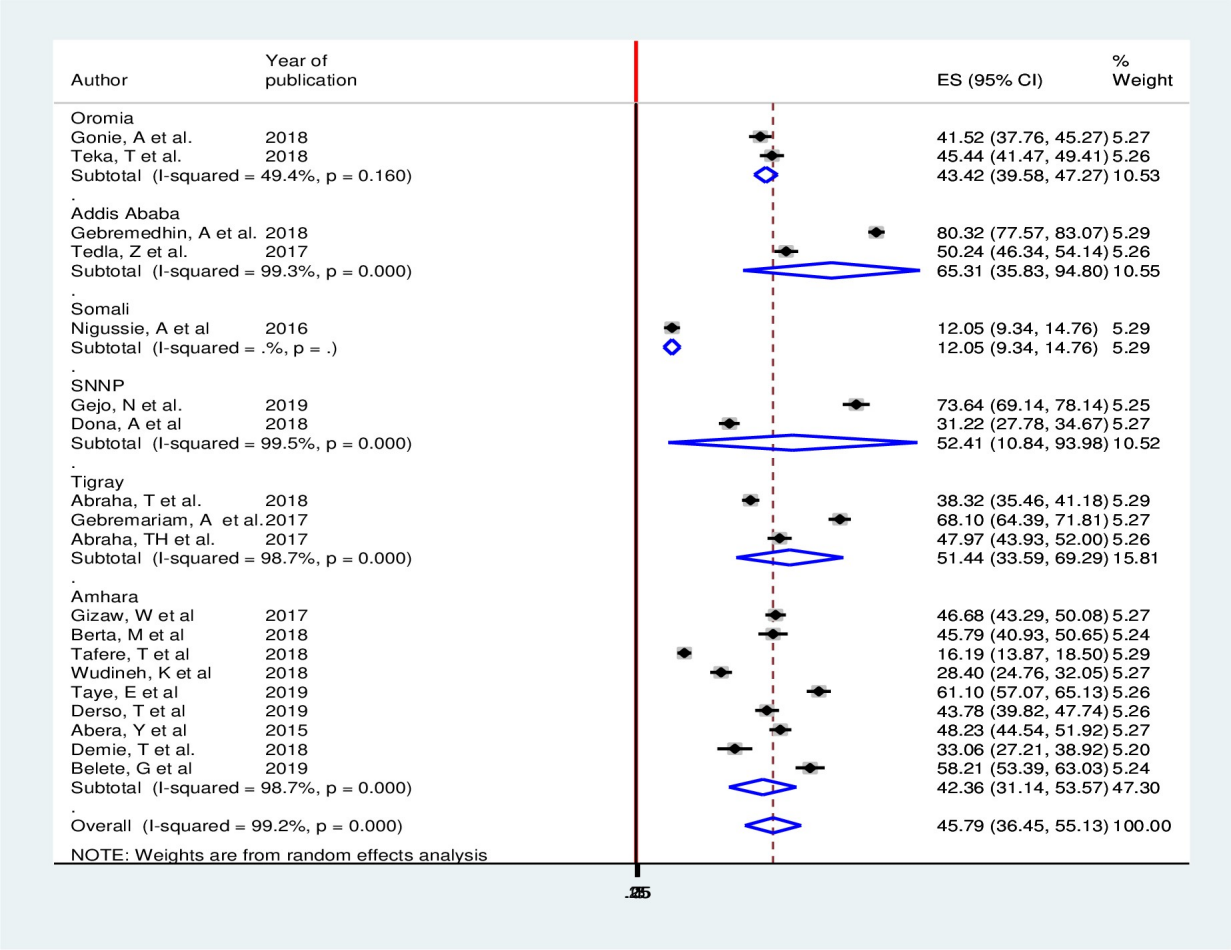
Forest plot subgroup analysis of magnitude of postpartum modern contraception utilization in Ethiopia, 2020.

Sub-group analysis was conducted based on the region in which the studies were done. Accordingly, the magnitude of postpartum modern contraception utilization is 12.05%(9.34%, 14.76%) in Somali [15] and 65.31% (35.83%, 94.80%) in Addis Ababa [14, 36] (Fig 3).

### Publication bias

**Fig 4:**
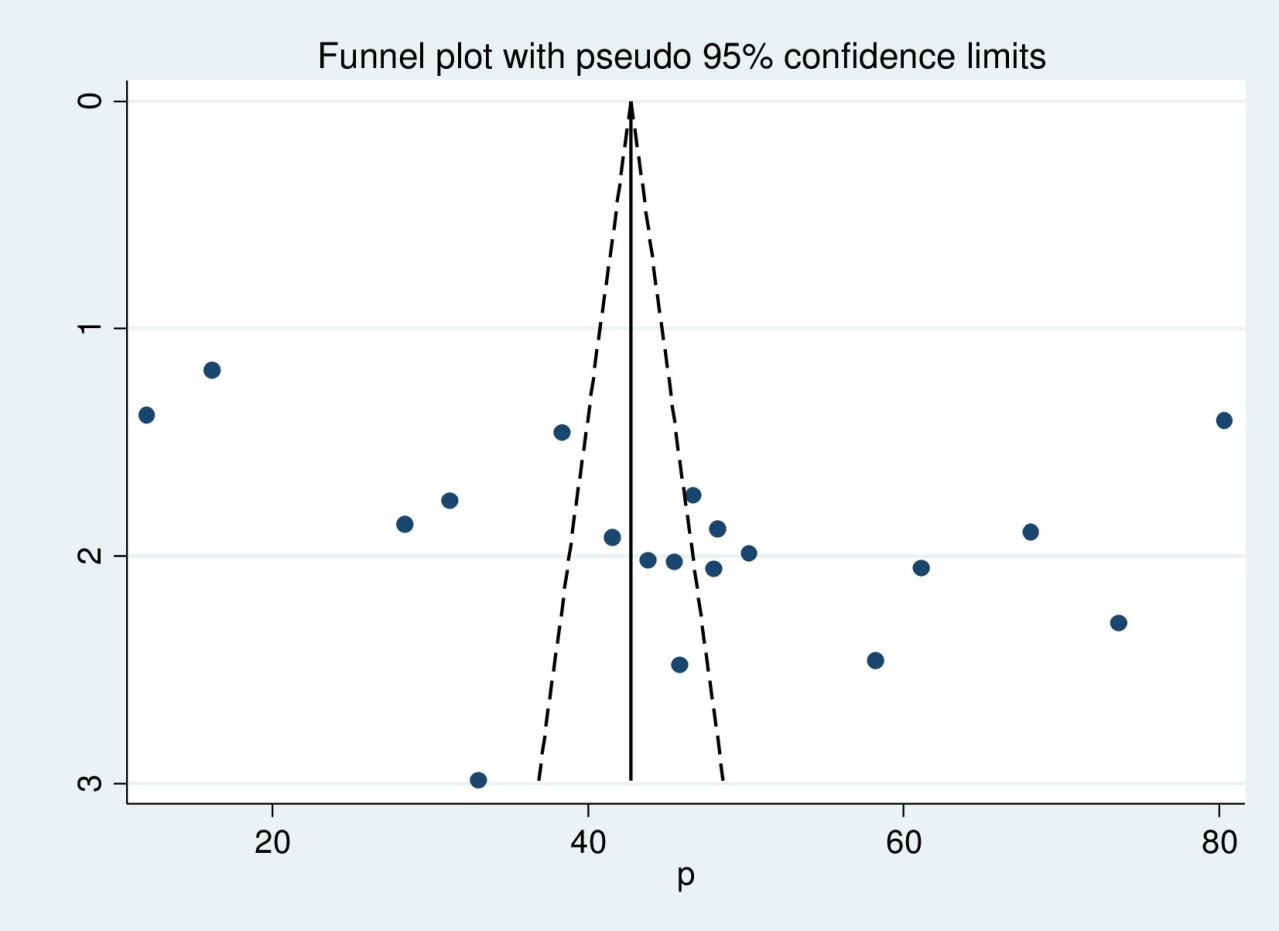
Funnel plot with 95% confidence limit of the pooled magnitude of postpartum modern contraception utilization in Ethiopia, 2020.

Publication bias was assessed by funnel plot and Egger test at a 5% significant level. The funnel plot was asymmetry, and the Egger test showed no statistical significance for the presence of publication bias with p-value, a P = 0.105 (Fig 4).

**Fig 5:**
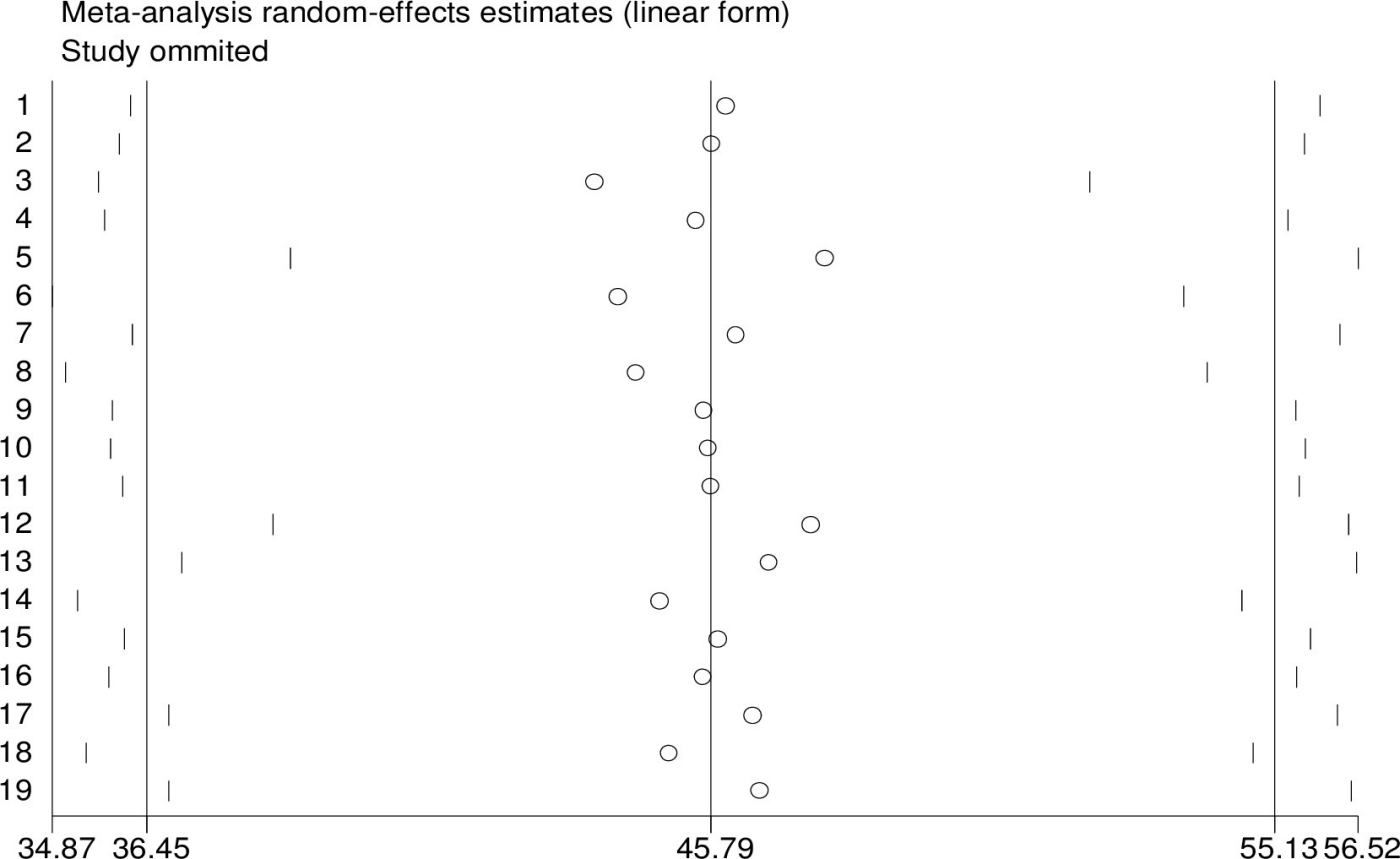
Sensitivity analyses for single study influence of magnitude of postpartum modern contraception utilization in Ethiopia, 2020.

The influence of a single study on the overall meta-analysis was checked using a random- effects model and the result showed there was no strong evidence for the effect of single study influence on the overall meta-analysis (Fig 5).

### Determinants of postpartum modern contraception utilization in Ethiopia

**Fig 6:**
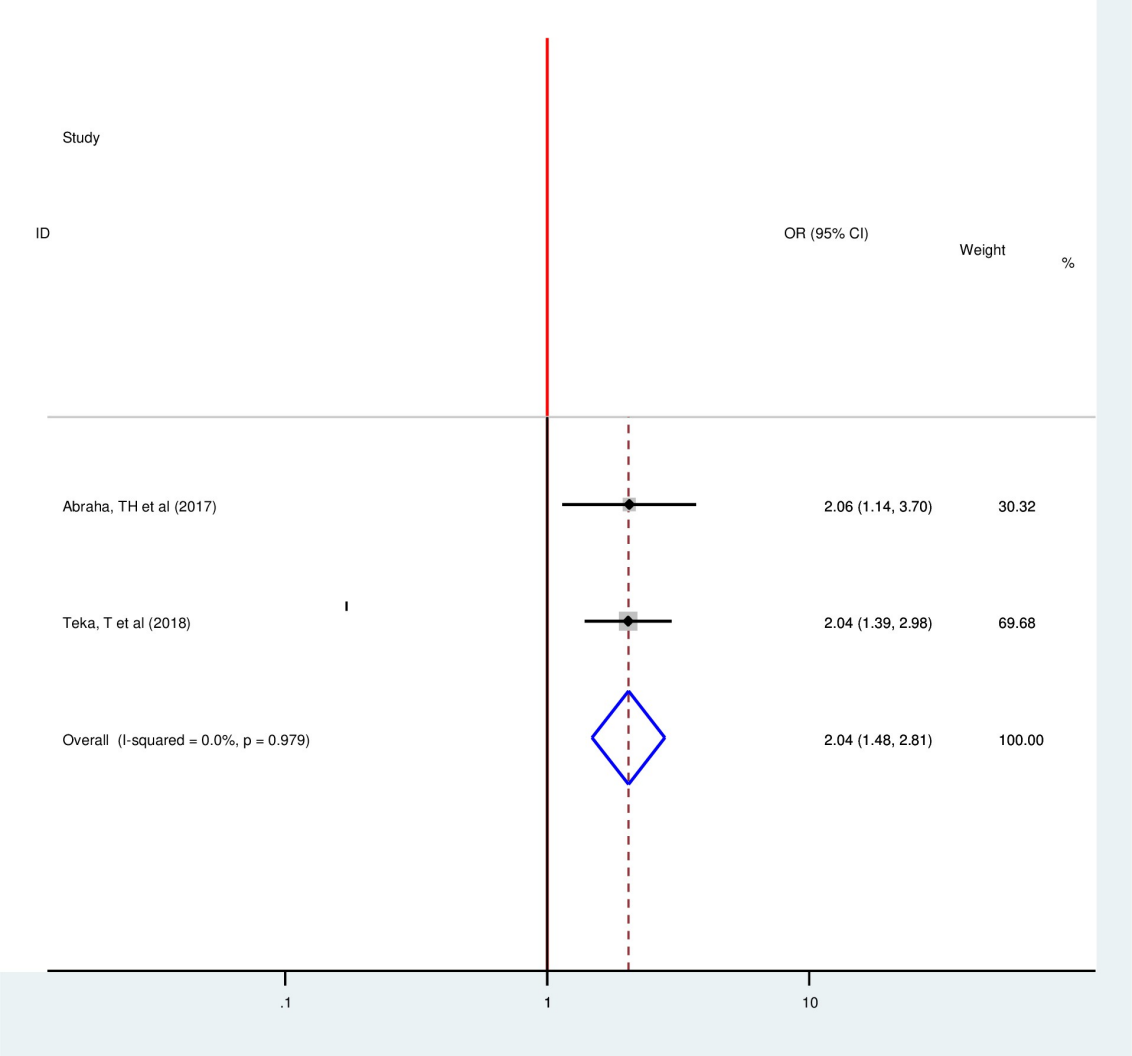
Forest plot of association between postpartum modern contraception utilization and number of ANC visit in Ethiopia, 2020.

**Fig 7:**
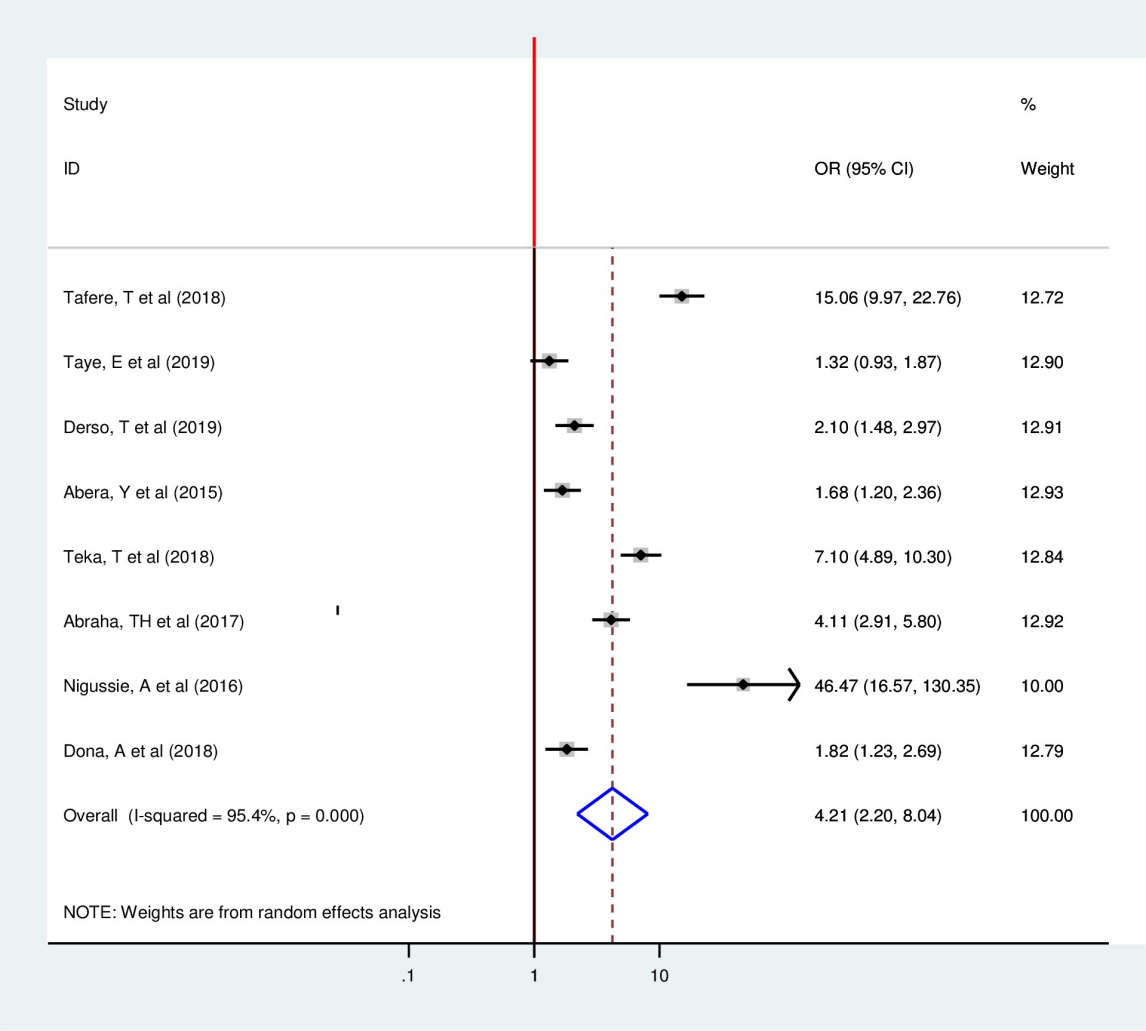
Forest plot of association between postpartum modern contraception and PNC visit among postnatal women in Ethiopia, 2020.

In this review, antenatal care visit, postnatal care visit, maternal education, counseling on family planning, previous family planning use, partner approval, and a number of alive children were strongly associated with postpartum modern contraception utilization. However, the number of parity and place of residence showed no statistically significant association with postpartum modern contraception utilization. Two articles showed that ANC visit was strongly associated with the postpartum modern contraception utilization [20, 33]. Those women who had four or greater ANC visits were 2.04 times more likely to utilize modern contraception as compared to their counterparts (OR = 2.04, 95%, CI: 1.48–2.81). Since the included studies were not exhibited heterogeneity fixed-effect model was used (I^2^ = 0.00%, p = 0.978) (Fig 6). Eight articles also revealed that PNC visit is positively associated with postpartum modern contraception utilization [15, 16, 18–20, 29, 33, 38]. Those women who had PNC visit were 4.21 times more likely to utilize postpartum modern contraception than their counterparts (OR = 4.2195%CI 2.20, 8.04) Random effect model was used for this Meta-regression (I^2^ = 95.4, P = 0.00) (Fig 7).

**Fig 8:**
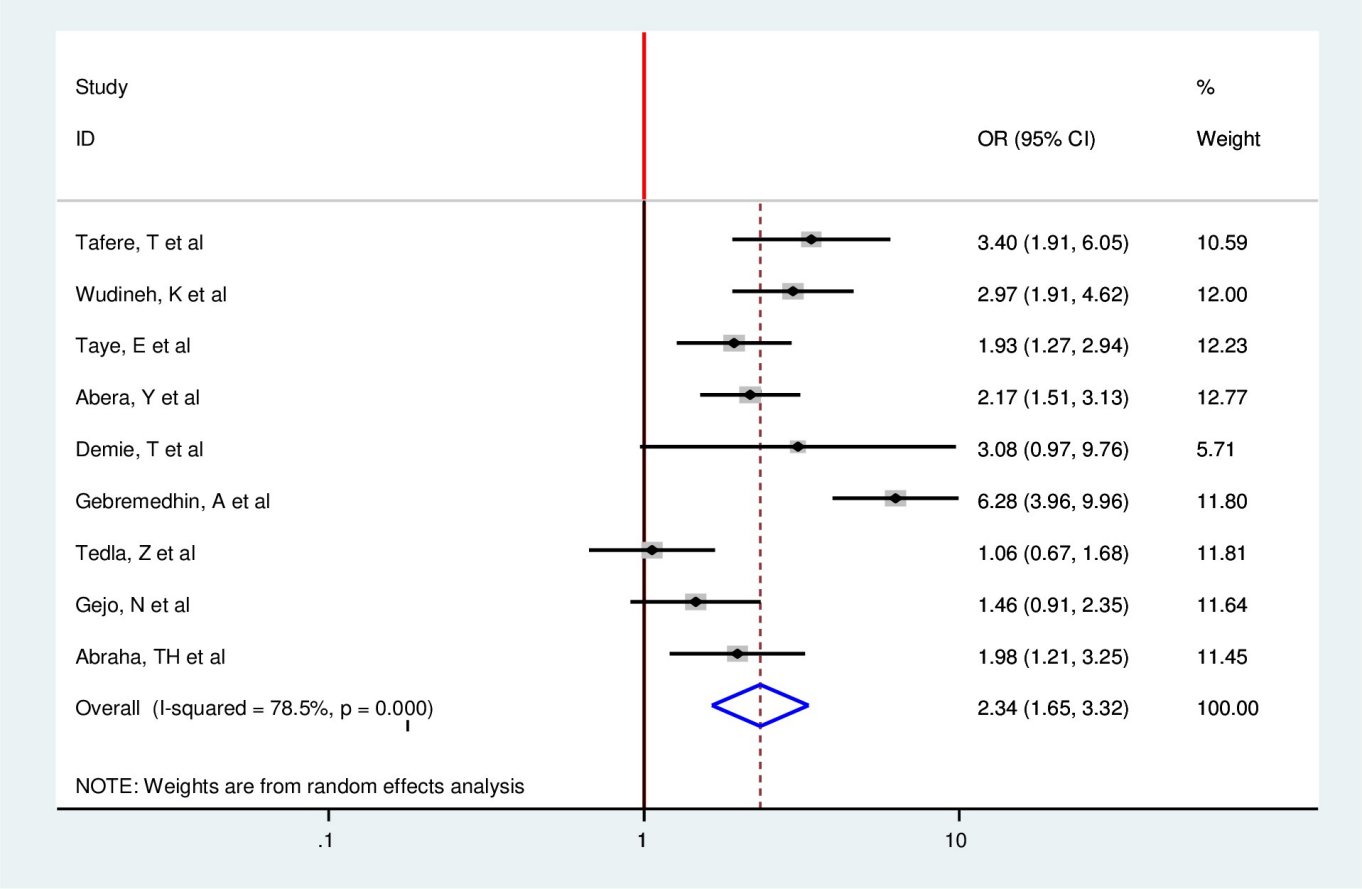
Forest plot of association between postpartum modern contraception utilization and educational status of the women in Ethiopia, 2020.

**Fig 9:**
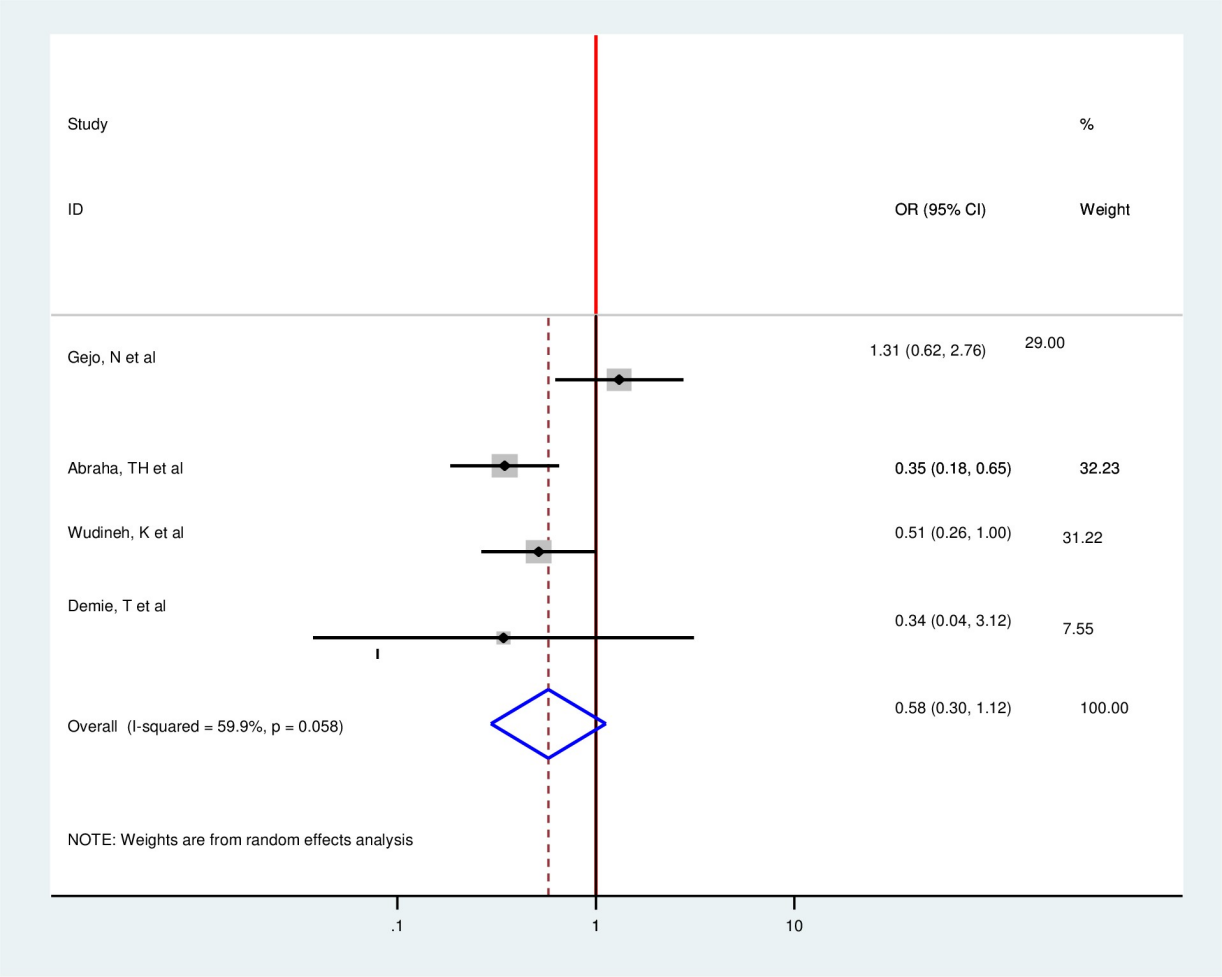
Forest plot of association between postpartum modern contraception utilization and number of parity among postnatal women in Ethiopia, 2020.

In addition, in this Meta-regression, six among nine included articles found a strong association between postpartum modern contraception utilization and the educational status of the women [14, 18, 19, 25, 33, 35, 36, 38]. Those women who have formal educations were 2.34 times more likely to utilize modern contraception in the postpartum period as compared with those who have no formal education (OR = 2.34 95%CI 1.65,3.32) [14, 18, 19, 25, 31, 38]. However, three articles showed no significant association with the outcome variable [33, 35, 36]. Since there is moderate heterogeneity, the random effect model was used (I^2^ = 78.5% P = 0.00) (Fig 8). Moreover, four article were assessed to find association between number of parity and postpartum modern contraception utilization [25, 31, 33, 35]. From the pooled finding, there was no significant assocaition between postpartum modern contraceptive utilization and number of parity among postpartum women (OR = 0.58 95%CI 0.30,1.12) by using the random effect model (I^2^ = 78.5,P = 0.00) (Fig 9).

**Fig 10:**
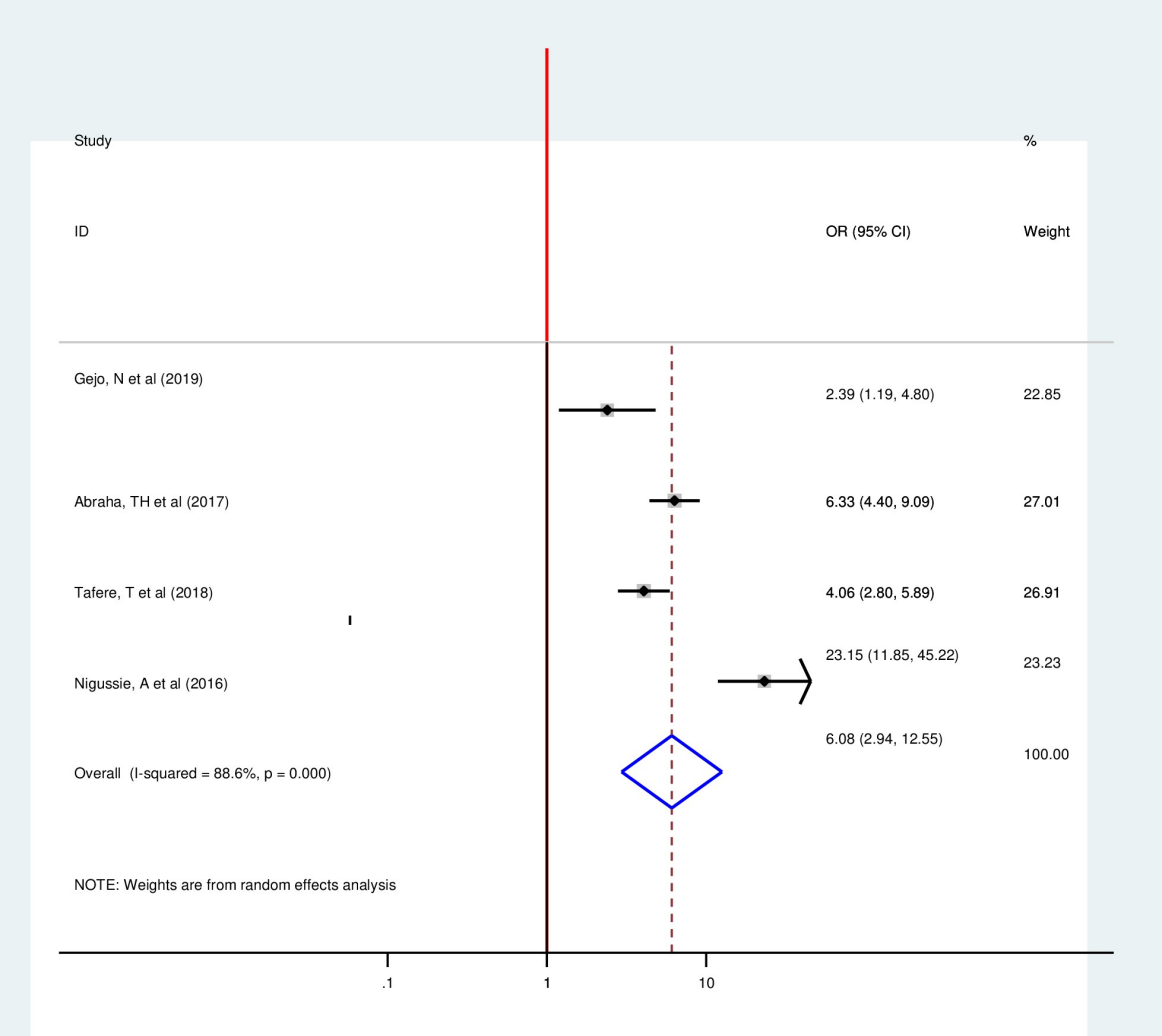
Forest plot of the association between postpartum modern contraception utilization and counseling on family planning among postnatal women in Ethiopia, 2020.

**Fig 11:**
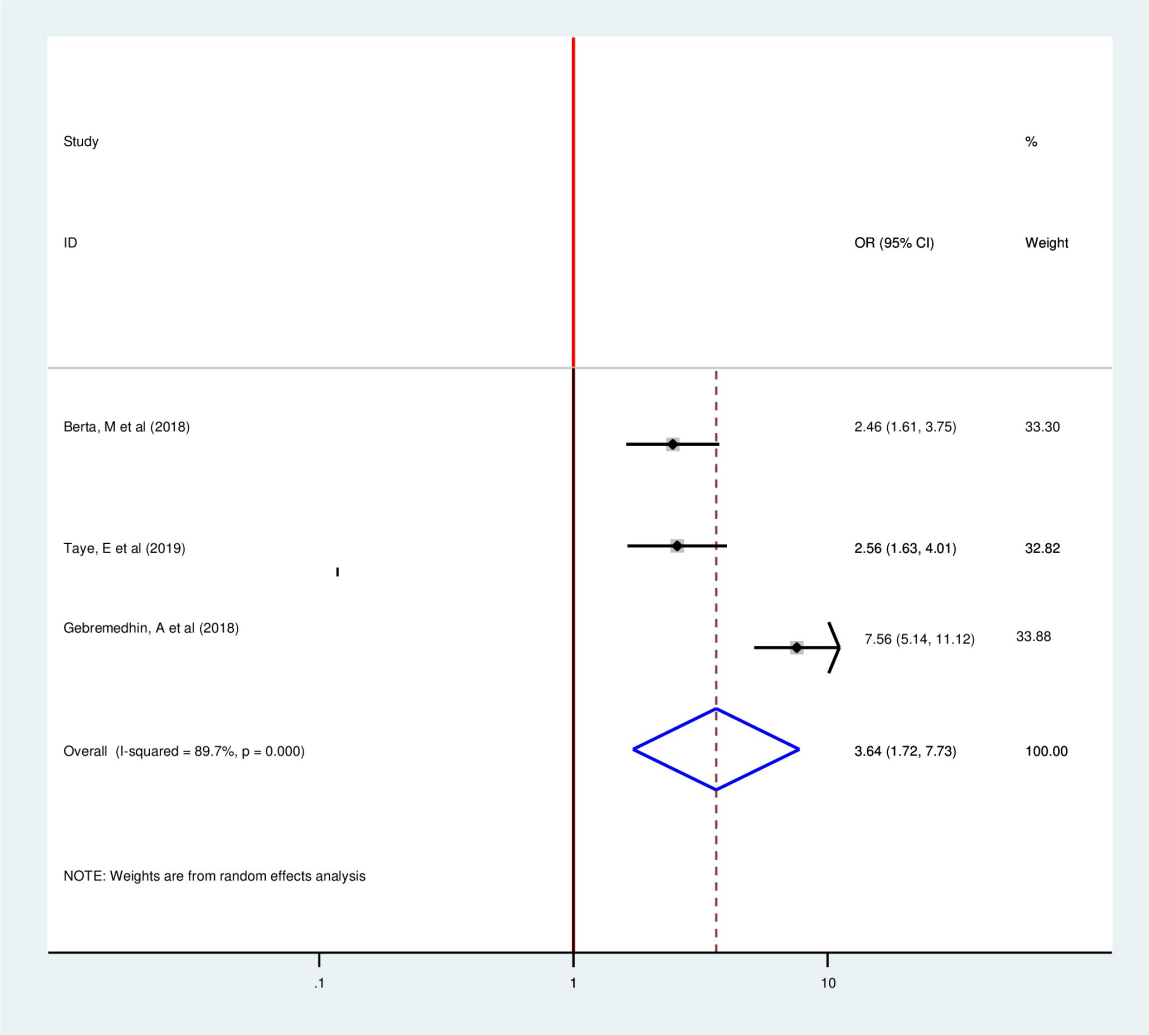
Forest plot of association between postpartum modern contraception utilization and history of previous family planning use in Ethiopia, 2020.

Four articles included in this Meta-regression also showed a strong association between counseling on family planning and postpartum modern contraception utilization [15, 18, 33, 35]. Consistent to the individual regression, those women who have counseling on family planning were 6.08 times more likely to use postpartum contraception as compared to their counterparts with (OR 6.08 95% CI 2.94, 12.55) by using random-effect model since there is heterogeneity (I2 = 88.6 P = 0.00) (Fig 10). This Review included three articles to determine the pooled effect of previous family planning use on postpartum modern contraception utilization and all of them showed significant association consistent with individual findings [14, 19, 28]. Postpartum mothers with history of previous family planning use were 3.64 times more likely to utilize postpartum modern contraception as compared to their counter parts with (OR 3.64, 95%, 1.72, 7.95)) by using random effect model (I^2^ = 89.7% P = 0.00) (Fig 11).

**Fig 12:**
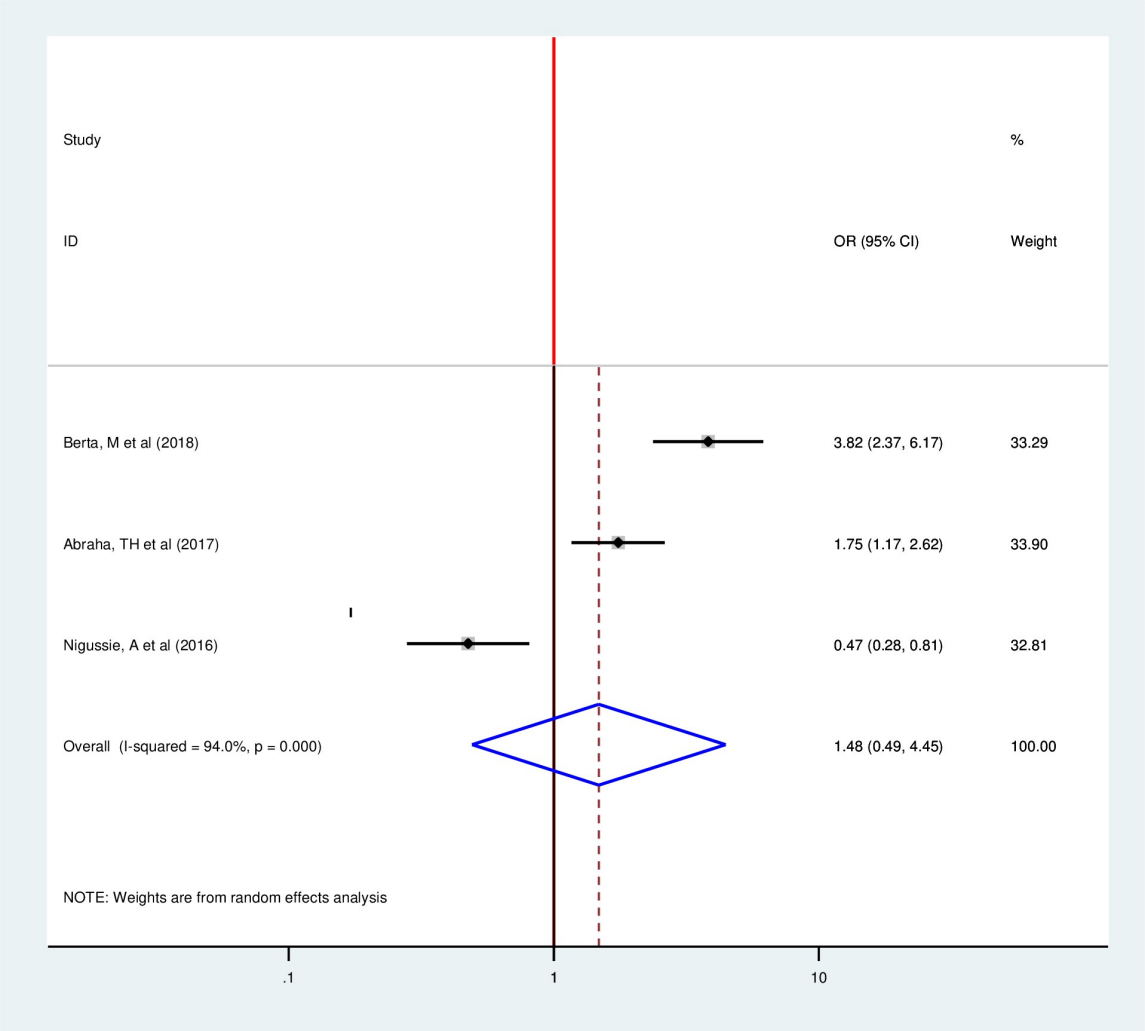
Forest plot of the association between postpartum modern contraception utilization and partner approval for family planning use among postnatal women in Ethiopia, 2020.

**Fig 13:**
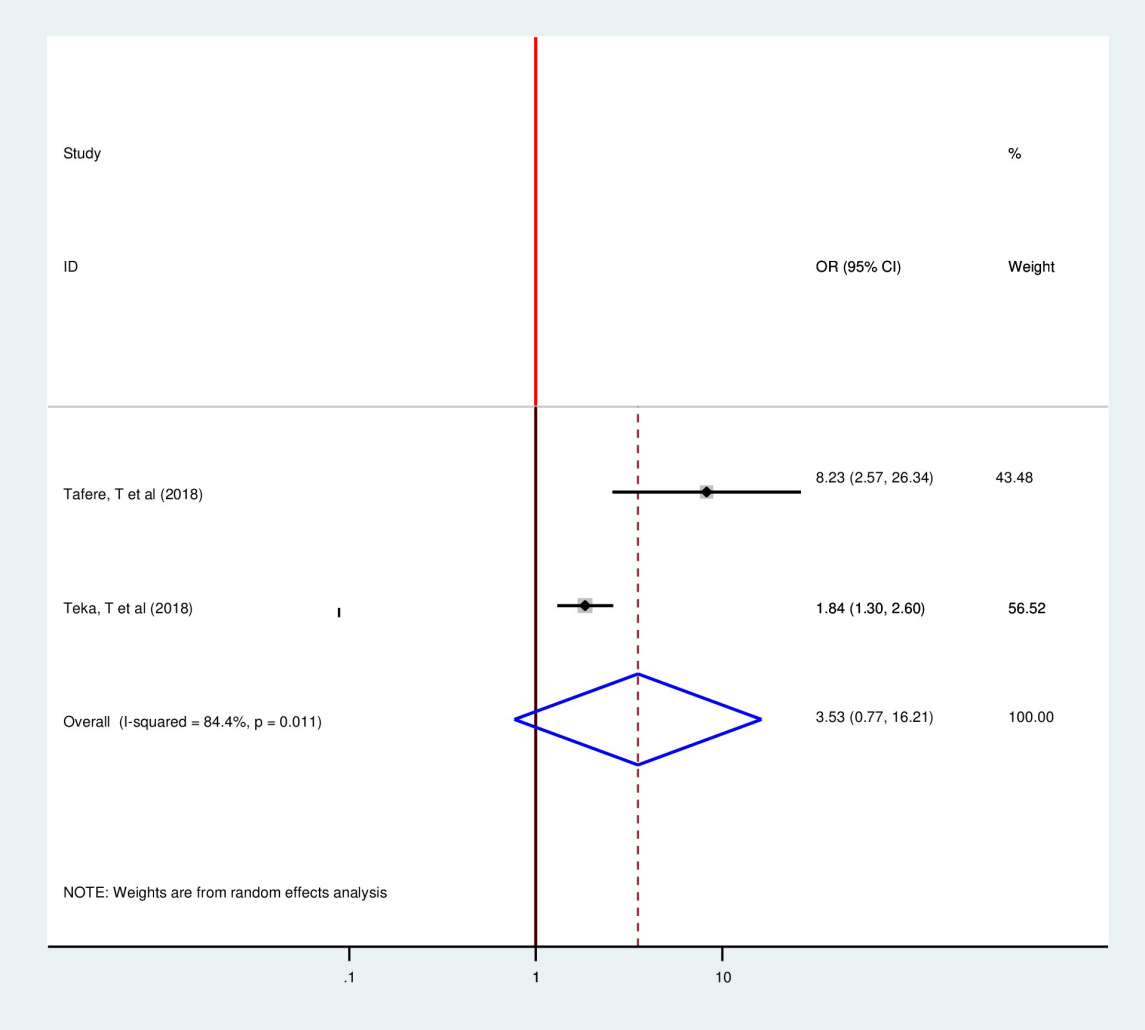
Forest plot of the association between postpartum modern contraception utilization and place of residence of postnatal women in Ethiopia, 2020.

This meta included three articles to determine the pooled effect of partner approval for family planning use on postpartum modern contraception utilization and all of them showed significant association as individual findings [15, 28, 33]. However, the pooled meta-regression showed that there is no significant association between partner approval and postpartum modern contraception utilization (OR 1.48, 95%, 0.49, 4.45)) by using the random-effect model (I2 = 89.7% P = 0.00) (Fig 12). This study included two articles to determine the pooled effect of residence on postpartum modern contraception utilization and both of them showed significant association as individual findings [18, 20]. However, the pooled meta-regression showed no significant association (OR 3.53, 95%, 0.77, 16.21)) by using the random effect model (I2 = 84.4% P = 0.011) (Fig 13).

**Fig 14:**
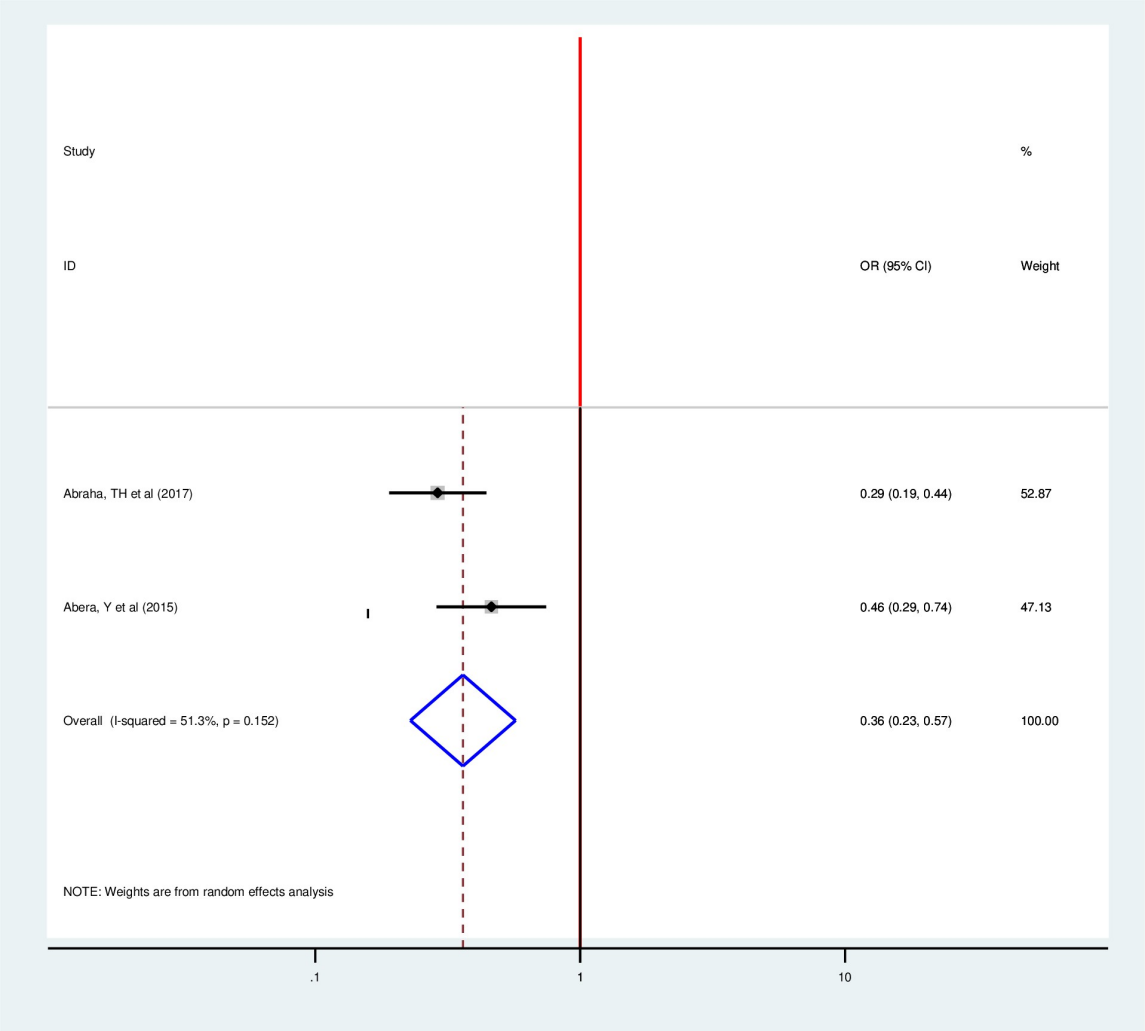
Forest plot of association between postpartum modern contraception utilization and number of alive children in Ethiopia, 2020.

The review included two articles to determine the pooled effect of the number of alive children on postpartum modern contraception utilization and both of them showed significant association consistent with individual findings [33, 38]. Thus, the probability of postpartum modern contraception use is 64% lower in postpartum women who have less than four alive children than their counterparts (OR 0.36, 95%, 0.23, 0.57)) by using the random effect model (I2 = 51.3% P = 0.152) (Fig 14).

## Discussion

Worldwide, maternal mortality continues to be a major public concern despite a variety of interventions that have been made [6]. Among which, solving the issue of unmet need for family planning among postpartum women was the strategy to end the preventable causes of maternal death. However, 70% of postnatal women in developing countries particularly Sub-Saharan Africa, are not using contraception though they do not want to conceive [11]. The aim of this review was to determine the best available pieces of evidence to pool the magnitude of postpartum modern contraception utilization and find out its determinants.

In this systematic review and meta-analysis, the magnitude of postpartum modern contraception utilization among postpartum women in Ethiopia was 45.79% (36.45%, 55.13%). This finding is high as compared to finding from Uganda (8.5%), India (13.8 to 17%), Benin (13%), and Burundi (20%) [39–43]. The reason for variation might be the larger sample size (11,932) in the current review while smaller sample size in the previous studies which ranges from 123 to 1830 [39, 40, 42, 43] and variation in the cultural background of the populations. However, the current study finding is low when compared to reports from Malawi (75%), Kenya (86.3%), Ghana (67.7%), and Bangladesh (62.4%) [44–47]. The discrepancy may be due to cultural influences and the relatively high unmet need for family planning in Ethiopia [5]. The present finding is comparable to a study finding from Ethiopia(48.11%) [17] and Rwanda (51.1%) [43].

In the present review, subgroup analysis by regions indicated that there were variations in the magnitude of postpartum modern contraception utilization among different included regions of Ethiopia. Accordingly, the highest postpartum modern contraception utilization was observed in Addis Ababa 65.31% (95% CI: 35.83, 94.80) [14, 36] followed by SNNP 52.41% (95% CI: 10.84, 93.98) [16, 35] while the lowest in Somali region 12.05% (95% CI: 9.34, 14.76) [15]. The variations observed might be due to cultural and religious differences among the regions. Moreover, it might be due to variation in access to the health care facility and information on the usefulness of contraception during the postpartum period.

In the current study, the educational status of the mother was positively associated with postpartum modern contraception utilization. Women who have formal education were 2.34(95% CI: 1.65, 3.32) times more likely to utilize modern contraception as compared to those who have no formal education. This finding is consistent with a study done in rural Kenya, Ghana in Talensi district, Bangladesh, and Uganda [45, 47–49]. This implies that education can improve women’s knowledge on contraception use which in turn minimize adverse outcomes of short interpregnancy outcome [1].

The present study also demonstrated that the probability of using modern contraception in the postpartum period is higher among counseled mothers on family planning 6.08 (95% CI: 2.94, 12.55) as compared to their counter parts. Moreover, this meta-analysis revealed that a history of previous contraceptive use increases the probability of contraception utilization in the postpartum period of 3.64(95% CI: 1.72, 7.73). A finding from a study conducted in Parakou Northern Benin supported this evidence [40]. This implies that counseling on family planning during antenatal & postnatal care has to get due attention and strengthened to overcome maternal and child health problems related to the short birth interval.

Existing evidence supported that post-delivery checkup positively influences postpartum modern contraception use [49]. Similarly, this study finding is in line with previous evidence that the likelihood of postpartum modern contraception use is 4.21(95% CI: 2.20, 8.04) times higher among women who have postnatal visit than their counter parts. This proves that encouraging postnatal care visits is an essential element of practice that would increase postpartum modern contraception utilization. In this systematic review and meta-analysis, the frequency of ANC visits was also found as a determinant factor of postpartum modern contraception use (95% CI: 1.48, 2.81). This evidence is supported by a study finding from Bangladesh that ANC visit was a predictor of postpartum modern contraception utilization [47]. This depicts that ANC service increases awareness of women on family planning and thus, improves contraception utilization.

In the present study, the likelihood of postpartum modern contraception use was 64% lower in postpartum women who have less than four alive children than their counterparts. Similarly, this evidence is supported by previous studies conducted in Burundi, Rwanda, and Uganda [39, 43]. This may be due to the fact that mothers who have less number of alive children rarely use contraception as they need to have more.

The finding of this review is important for policymakers and program planners in designing appropriate interventions to improve the problem related to modern contraceptive utilization of postpartum women. The study assessed limited factors associated with postpartum modern contraceptive utilization, but factors associated with postpartum contraceptive utilization are not limited to these variables. So, the finding of this study guides researchers to conduct large scale and qualitative study to see the relationship between postpartum contraceptive utilization and other factors not included in this analysis.

### Strength and limitations of the study

In this review, not only published but also unpublished articles were extensively searched using different databases. Moreover, the review protocol was registered on Prospero, an International prospective register of systematic reviews to avoid duplication of systematic review and meta-analysis on a similar topic. However, this review is not without limitations as the primary studies included in this review were limited to some regions of the country, other regions might be underrepresented. Only articles published in the English language were considered to conduct this review, which may result in missing studies that could have been published in other languages. Moreover, there were few primary studies with small a sample size included in the review which might influence the estimated magnitude of postpartum modern contraception use. Finally, almost all of the studies included in this review were cross-sectional study designs which decreases the causal relationship between postpartum contraceptive utilization and its determinants.

## Conclusion

The magnitude of postpartum modern contraception utilization in Ethiopia is low. ANC visit, PNC visit, maternal educational status, previous family planning use, counseling on family planning, and a number of alive children were found to be significantly associated with postpartum modern contraception utilization in Ethiopia. Therefore, strengthening adherence to focused ANC utilization and PNC services should be given due attention to encourage women in utilizing modern contraception during the postnatal period. Furthermore, scaling up women’s educational status has paramount importance in increasing postpartum modern contraception use which further improves maternal and child health in general.

## Supporting information

S1 Checklist PRISMA 2009 checklist. (DOC)

S1 Dataset (XLSX)

## Abbreviations

ANC: Antenatal care
AOR: Adjusted Odds ratio
CI: Confidence Interval
FP: Family planning
IUCD: Intrauterine contraceptive device
JBI-MAStARI: Joanna Briggs Institute Meta-Analysis of Statistics Assessment and Review Instrument
PNC: Postnatal care
PRISMA: Preferred Reporting Items for Systematic Review and Meta-Analyses
SDG: Sustainable development goal
WHO: World Health Organization
